# Extrachromosomal circular DNAs: an extra piece of evidence to depict tumor heterogeneity

**DOI:** 10.2144/fsoa-2019-0024

**Published:** 2019-05-31

**Authors:** Ishita Tandon, Roshni Pal, Jayanta K Pal, Nilesh K Sharma

**Affiliations:** 1 Cancer & Translational Research Lab, Dr DY Patil Biotechnology & Bioinformatics Institute, Dr DY Patil Vidyapeeth, Pune, Maharashtra 411033, India

**Keywords:** biomarker, DNA repair, eccDNAs, epigenome, neoplasms, tumor heterogeneity, tumor microenvironment

## Abstract

The tumor microenvironment (TME) comprises a heterogeneous number and type of cellular and noncellular components that vary in the context of molecular, genomic and epigenomic levels. The genotypic diversity and plasticity within cancer cells are known to be affected by genomic instability and genome alterations. Besides genomic instability within the chromosomal linear DNA, an extra factor appears in the form of extrachromosomal circular DNAs (eccDNAs; 2–20 kbp) and microDNAs (200–400 bp). This extra heterogeneity within cancer cells in the form of an abundance of eccDNAs adds another dimension to the expression of procancer players, such as oncoproteins, acting as a driver for cancer cell survival and proliferation. This article reviews research into eccDNAs centering around cancer plasticity and hallmarks, and discusses these facts in light of therapeutics and biomarker development.

## Lay abstract

Currently, cancer patients are faced with difficulties with early detection, high doses of drugs, insufficient monitoring of drug efficacy, drug resistance and relapse. Tumor heterogeneity including genetic and extrachromosomal factors is known to be linked with various cancer-related problems. The presence and abundance of extrachromosomal circular DNAs in cancer patients could be harnessed to aid early detection of cancer, as well as for development of new classes of drugs/inhibitors.

Recently, appreciable attention has been drawn to the understanding and harnessing of tumor heterogeneity in the context of preclinical and clinical aspects of cancer biomarkers and therapeutics [1–5]. Tumor heterogeneity is known to be affected by several factors such as molecular, genetic and epigenetic changes, extrachromosomal circular DNAs (eccDNAs), nuclear microDNAs, secreted microDNAs, noncoding miRNAs and environmental pressures [1–9]. It is widely known that genetic aberrations and instabilities at the genome level in several forms drive carcinogenesis, as well as several key tumor hallmarks and adaptations within the tumor microenvironment (TME) [3–9]. Therefore, basic, preclinical and clinical studies are required to seek the change of conducive pro-TME to an anticancer microenvironment, ensuring the success of next-generation therapeutics such as precision and personalized medicine [3–9].

In addition to genomic alterations in linear chromosomal DNA, the contribution of eccDNAs to some extent is envisaged as regards observed tumor heterogeneity and crucial tumor hallmarks such as invasiveness and drug resistance [8–19]. The existence of eccDNAs in normal cells as well as cancer cells was demonstrated a few decades ago. The origin of eccDNAs within the nucleus is facilitated by normal cellular processes, an abnormal DNA repair system and environmental pressures such as stress, carcinogens and pathogens [12–28]. Interestingly, these eccDNAs are reported in several organisms including *Drosophila*, yeast, plants, animals and humans [8–12,29–34]. However, abundance of these eccDNAs is a matter of investigation across different organisms and between normal and cancer cells. Hence, attempts have been made to understand the mechanisms of formation of eccDNAs in different types of cells such as normal cells, cancer cells, and cancer-associated cells and distinct cellular heterogeneity within a tumor [8–12,29–34].

Current focuses on eccDNAs are also inclined toward understanding ways to mitigate their abundance and tumorigenic impact within the cellular heterogeneity of a tumor. Various potential avenues are suggested in the form of gene therapy, small RNA mimetics to interfere with eccDNAs, natural sources of small RNAs and inhibitors of the DNA repair protein system [35–40]. Furthermore, evidence from eccDNA research is being translated into avenues for development of cancer biomarkers and diagnostic tools [35–40]. This mini-review addresses the recent developments in deciphering a link between eccDNAs and TME that may be translated into future therapeutic and diagnostic avenues.

## Tumor heterogeneity driving tumor hallmarks

**Figure 1.**
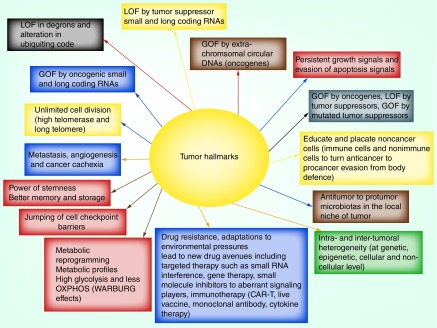
Tumor hallmarks. The updated tumor hallmarks that define the true nature of complexity and heterogeneity of a tumor, encompassing deregulations at various levels including molecular, genetic, epigenetic, eccDNAs, microDNAs, secreted microDNAs and noncoding miRNAs and environmental pressure. CAR-T: Chimeric antigen receptor-T cell therapy; GOF: Gain of function; LOF: Loss of function; OXPHOS: Oxidative phosphorylation.

Recently, the complex nature of cancer has been attributed to the tumor heterogeneity emanating at different levels including at the molecular, genomic and epigenomic level, and various environmental pressures [1–9,41–44]. In a true sense, tumor heterogeneity that drives growth, proliferation and invasiveness is essentially additive in nature [1,2,9,43,44]. With reference to genetic heterogeneity, research has established that aberrations and variations at the gene level within the linear chromosomal DNAs drive tumor heterogeneity in the form of procancer factors [8,12,13,15–19,25]. However, there is limited evidence demonstrating the implications of increased number of eccDNAs (2–20 kbp size) and also circular microDNAs (200–400 bp) within the nucleus of cancer cells [8–12,15–19,25,33,34]. In brief, heterogeneity displayed by various factors including genetic, epigenetic, eccDNAs, nuclear microDNAs, secreted microDNAs, miRNAs and environmental pressures contributes toward complexity and plasticity within a tumor. A summary of updated tumor hallmarks is presented in Figure 1.

## Extrachromosomal circular DNAs

In living organisms, the cellular landscape is known to have distinct genetic components including chromosomal nuclear DNA, coding RNAs, noncoding regulatory RNAs and so on. Besides chromosomal nuclear DNA, eccDNAs are reported in cytoplasmic organelles such as mitochondria in animal cells and both mitochondria and chloroplasts in plant cells [10,11,17,20,29–32].

Besides the mitochondrial and viral origin of eccDNAs, eccDNAs are present in the nucleus of normal and unhealthy cells (including cancer cells), and are clearly linked to coherent cellular signaling and functions [12,14,17–19,24–28]. These specialized eccDNAs are mostly suggested to originate from repetitive genomic sequences such as telomeric DNA or rDNA [1,8,12–19,25,34].

Various organisms including yeast, *Caenorhabditis elegans*, *Drosophila melanogaster*, mammals and plants have been shown to harbor eccDNAs in the nucleus and their presence is linked to normal functions and associated phenotypes of these organisms [8,10–20,25,29–34]. Another observation indicates the complexity of small circular DNA in *D. melanogaster* and also the homology between small circular DNA and middle-repetitive chromosomal DNA. These small circular DNAs are shown to possess mobility which can result in deletions, mutations, chromosomal variations and oncogenesis [29]. There are contrasting differences in the description of the basis of generation of eccDNAs in eukaryotic cells. One study proposes that in the case of *Drosophila*, eccDNAs are formed by a preferred homologous recombination process between tandem repeats, and this study further suggested that formation of eccDNAs is independent of any DNA repair process [33]. Meanwhile, another study on Xenopus suggests that *de novo* synthesis from naked DNA and telomere DNA of sperm nuclei results in the development of extrachromosomal circular telomeric DNA consisting of vertebrate repeats of telomeric DNA (TTAGGG)n [11]. Previous research screening yeast genomic DNA indicated that eccDNAs are found in abundance (~23%), which led to the claim that eccDNAs are one of the factors involved in mutation and evolutionary characteristics of the eukaryotic genome [12].

Interestingly, the contribution of nonrepetitive DNA to the rise of eccDNAs is drawing wide attention in the case of both normal and cancer cells [1,2,5,8,10,12–19,25]. Shoura *et al.* presented evidence in support of the presence of eccDNAs in *C. elegans* and human-derived cell lines [18]. Data indicate that eccDNAs can originate from both coding and noncoding regions in response to normal cellular settings. It is important to mention that in prokaryotic and eukaryotic cells, besides the main genome-contributing chromosome, small circular double-stranded DNA such as eccDNAs originate from repetitive DNA sequences within the genome (2–20 Kbp) and serve various purposes including drug resistance, cellular survival, metabolism, signaling and death [12,14,17–28]. Currently, approaches such as Circle-Seq, 2D gel electrophoresis and next-generation sequencing are reported to validate the presence of eccDNAs in various eukaryotic cells including yeasts, plant cells and animal cells. Taken together, cutting edge genomics tools clearly support the presence of eccDNAs in various cell types including of the mammalian system and these results advocate further exploration to find clues and linkages for normal developmental processes and disease conditions including tumors.

## Mechanisms of formation of eccDNAs

In both normal and cancer cells, formation of eccDNAs is suggested to be both by DNA replication-dependent and -independent means [12,14,17–19,22–28]. Commonly, eccDNAs from organisms ranging from yeasts, to plants and to animals are suggested to originate from long-repetitive, short-repetitive and nonrepetitive sequences of the genome [14,17,33].

In the majority of cases, these eccDNAs are known to contain ribosomal genes, transposon remnants, coding tandemly repeated genes (HXT6/7, ENA1/2/5 and CUP1-1/-2), noncoding chromosomal high-copy tandem repeats, telomeric DNA and oncogenes [12,14,17–19,22–28]. Recently, Shibata *et al.* convincingly suggested the existence of eccDNA as microDNAs in the range of 200–400 bp [25]. Their data suggest that these eccDNAs can originate from unique nonrepetitive DNA sequence in mouse tissues as well as mouse and human cell lines.

The eccDNAs or ring chromosomes are suggested to cause deletions, mutations, replications, amplifications or translocations of genes; these genetic alterations are attributed to eccDNAs for their uniqueness in changing size, and self-deletion and integration within the genome [12,14,17–19,28,45]. As described earlier, evidence was found on *de novo* synthesis of eccDNAs in Xenopus embryos, which suggested that eccDNA formation is not a random DNA replication between maternal tandemly repeated multimers and the paternal genomic DNA template, but is a *de novo* process unique to the preblastula stage of development [10].

In contrast to the idea of *de novo* synthesis and a DNA replication-dependent process of formation for eccDNAs, another study suggests that their origin requires excision of chromosomal sequences facilitated by sequence independent enzymes independent of ATP solely with the help of Mg^2+^ and ameliorated through the double-strand DNA break repair system [24].

Recently, microDNAs ranging from 200–400 bp have been reported to originate mostly from the nonrepetitive sequences within the genome as a result of heightened abnormalities in the DNA repair system including homologous recombination, nonhomologous recombination and the mismatch repair system [12,14,17–28]. However, little is known about the mechanisms inducing the alterations of copy number of eccDNAs within the nucleus of animal, yeast and plant cells.

Additional views are accumulating to indicate the existence of DNA-independent mechanisms involving the DNA repair system to support the enhanced presence of eccDNAs in cancer cells [12,14,17–19,26–28]. In research supporting the basis of elevated levels of eccDNA in mammalian cells, Cohen *et al.* showed that extrachromosomal circular major satellites can be a hotspot for the generation of eccDNAs [23]. Their data also suggest the contribution of DNA replication and DNA ligase IV toward generation of eccDNA in highly proliferating cancer cells. As an additional basis of eccDNA generation, data indicate that defective MSH3 DNA mismatch repair protein can result in decreased levels of eccDNAs emanating from the non-CpG regions during normal cellular physiology [26]. Another finding reports on the presence of a varied size of cell-free eccDNAs, from 31–19,989 bp, and showed a higher GC content in case of microDNAs less than 500 bp. Furthermore, the formation of these eccDNAs is linked to the non-homologous end joining mechanisms of the DNA repair pathway [38]. Taken together, these eccDNAs are suggested to be present in both normal and abnormal cells. Currently, eccDNAs are being studied for their role in genetic variation, evolution, genomic instability, mutation and tumorigenesis.

## Tumor hallmarks & eccDNAs

In nature, the eukaryotic system shows unique developmental patterns, alterations in phenotype due to environmental cues, various types of stress, aging and chemotherapeutic cancer drug resistance. These processes are associated with the high abundance of eccDNAs [4,19,32,45–47]. Recently, it has become apparent that tumor hallmarks are driven by various factors including genetic, epigenetic and environmental pressures. At the genetic level, chromosomal and extrachromosomal components of nuclear and mitochondrial origin have been shown to contribute toward tumor heterogeneity [13,18,19]. These extra players are commonly found in various tissues and cell types, and in both normal and diseased conditions. Due to their highly heterogeneous origins and widespread occurrence in nearly all eukaryotes, eccDNAs are believed to reflect the genome’s plasticity and instability [4,19,37,45–47]. The significance of eccDNAs in eukaryotic DNA amplification associated with cancer development and metastasis has been studied [31].

Additionally, another experiment provides an insight into another possible approach – that submicroscopic elements comprising human MYC oncogenes that replicate semiconservatively serve as a precursor of double-minutes [48]. Sen *et al.* reported on the successful lysis of colon carcinoma cells to detect the submicroscopic structures of eccDNAs by alkaline lysis and PCR [49].

An earlier study suggests the presence of eccDNAs in HeLa S3 cells, which could have originated due to nonhomologous recombination within the nonrepetitive or low-copy DNA sequences [50]. Hence, rejoining fragmented DNA could be a step to generate eccDNAs in the cellular system. It is true that under different circumstances, healthy and unhealthy cells contain and release extracellular free eccDNAs of variable size, sequence complexity, copy number and homology to chromosomal DNA [4,19,37,45–50]. These variations in eccDNAs – specifically with reference to tumor tissues – are attributed to several factors including genotype of the tumor, intratumor heterogeneity and environmental factors including lifestyle, nutrition, stress and so on [4,19,37,45–51]. A significant research analysis in glioblastoma of approximately 198 patients has shown the prevalence of amplification-linked extrachromosomal mutations due to which there are momentous mutations in oncogenes such as PDGFRA or EGFR in glioblastoma due to environmental effects [52]. A recent report describes the presence of microDNAs, a class of eccDNAs, in 20 independent human lymphoblastoid cell lines in the context of treatment of chemotherapeutic drugs [47]. In this experiment, their findings confirmed the generation of 190 bp microDNAs from the active regions of the genome and drug treatment demonstrated an increased proportion of microDNA loci in human lymphoblastoid cell lines. Interesting evidence has been found using whole-genome sequencing that detected extrachromosomal DNA in approximately half the human cancers and varied in frequency in each cancer type.

Recent whole-genome sequencing data on 17 different cancer types showed the presence of eccDNAs in nearly half of human cancers [4]. Furthermore, these data indicate that frequency of eccDNAs may vary by tumor type. In this study, the authors supported the role of eccDNAs in the acceleration of tumor progression and malignancy.

Despite valid questions regarding the possible origin and nature of the DNA sequence, nature of function and clinical relevance, the existence of eccDNAs is attracting an appreciable place in the area of cancer biomarkers and therapeutics [19].

## Contribution of eccDNAs in drug resistance

It has been suggested that the eccDNAs derived from a single cancer cell or heterogeneous cancer cells intensify the complexity of the resultant tumor, displaying drug resistance [1,35–40]. One such drug, Methotrexate, an antimetabolite drug, has been shown to induce drug resistance in colon cancer cell lines and the observed drug resistance is linked to the presence of intrachromosomal homogeneously staining regions or double minutes [28]. Another report suggests the role of extrachromosomal plasmid DNA to confer drug resistance in cervical cancer [52]. Overall, the driving forces behind cancer drug resistance, propelled by the presence of eccDNAs, are as yet unclear and in future focused studies are warranted to uncover these issues to aid in cancer therapeutics.

## Clinical scope of eccDNAs

Recently, detailed scientific evidence has support the emergence of new forms of tumor hallmarks including amplification of chromosomal and extrachromosomal circular oncogenes, and oncogenic noncoding miRNAs [1,2,4,6–9,35,41–44]. Understanding the underlying mechanisms of eccDNAs in tumor heterogeneity, evolution, development and metastasis has led to diverse clinical strategies for conquering cancer [1,38–40]. A new therapeutic approach can be devised by targeting inactivation of amplified copies of eccDNAs harboring oncogenes by using gene therapies, small RNA-based interferences and mimetic technology [1,35–40]. A scientific model has evolved to reveal that microtubule-mediated antipolar force can successfully tether chromosome to increase mitotic segregation of eccDNAs. Therefore, drugs targeting microtubule formation could be helpful in mitigating the abundance of eccDNAs in cancer cells [21]. Furthermore, the use of tyrosine kinase inhibitors (TKIs) could be useful to eliminate glioblastoma and low-grade gliomas displaying the presence of mutations in double minutes copies [52]. Another study has demonstrated that drug resistance to TKIs targeted to epidermal growth factor receptor is linked to maintenance of mutant EGFR in cancer cells with highly abundant copies of eccDNAs [46].

The observed differences between normal and cancer cells in terms of extent and nature of eccDNAs (2–20 kbp) and eccDNAs in the form of microDNAs (100–400 bp) are suggested to serve as biomarkers in cancer research [1–4]. These eccDNAs and microDNAs are shown to be released as extracellular free DNAs in the tumor. These can then serve as a basis for detection, monitoring of tumor responses to drug treatment and survival [35–40]. It is also proposed as the early biomarkers in certain tumor types detected as extracellular free eccDNAs in tumor tissues and other biological fluids of cancer patients [35–40].

**Figure 2.**
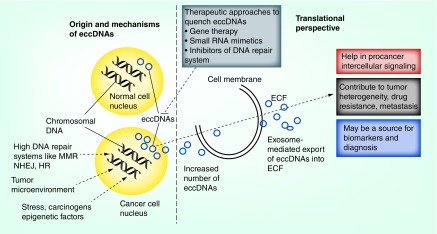
Extrachromosomal circular DNAs: origin, mechanisms and use as potential cancer biomarkers and therapeutic agents. Various factors in the form of stress, carcinogens, the DNA repair system, and normal cellular process such as DNA replication and epigenetic processes that contribute to the eccDNA heterogeneity within a tumor, are depicted. Potential cancer therapeutic avenues in the form of gene therapy, small RNA mimetics and inhibitors of DNA repair system are also highlighted. eccDNA: Extrachromosomal DNA; ECF: Extracytoplasmic function; HR: Homologous recombination; MMR: Mismatch repair; NHEJ: Nonhomologous end joining.

In summary, cell-free circulating circular tumor DNAs are presented as a new dimension to the existing tools of use, which include cell-free circulating linear DNAs and miRNAs in tumor clinical samples including tumor tissue, plasma, serum and urine. It would be exciting to see correlations among these cell-free small molecules of interest in the context of intra- and interheterogeneity of cancer. With the assistance of next-generation sequencing, more eccDNAs have been characterized at the molecular level. Recently, eccDNAs have been reported as cell-free DNAs in the circulatory system [35–40]. Importantly, these circulating eccDNAs have shown some disease associations, suggesting their potential utility as a new type of biomarker for disease detection, treatment assessment and progress surveillance. However, many challenges need to be addressed before implementing eccDNAs as a new source of genetic material for liquid biopsy [39]. Taken together, there are expanding horizons for evaluation of the basis of formation of eccDNAs, and the mechanisms adopted by eccDNAs to contribute toward tumor heterogeneity as cellular and noncellular secreted factors. Promising avenues are envisioned for these extra players within the nucleus of cancer cells, for them to become major partners to achieve therapeutic and diagnostic goals in the future (Figure 2).

## Future perspective

Besides the above described role of eccDNAs in tumor heterogeneity, little is known about the role of environmental pressures within the tumor that lead to the generation of eccDNAs and their shuttling from nucleus to cytoplasm and potentially to the extracellular milieu. Here, we speculate that these eccDNAs serve as messengers from primary to metastatic tumor sites by transferring the key driver genes for metastasis and the formation of secondary tumor. Further, we support the very recent observation that primary tumor actually dictates the formation of TME and subsequent tumor progression at secondary sites by employing eccDNAs as a cargo to deliver key genetic information. In other words, metastatic secondary tumor formation is contingent upon the delivery of information from primary tumor sites in the form of several factors including eccDNAs. Attempts to reveal variations of eccDNAs at the intra- and intertumor heterogeneity level is recommended that will help in future precision and personalized cancer therapy. Furthermore, the use of eccDNAs as a source of early detection biomarkers in the extracellular tissue fluid and other biological fluid sources including serum, saliva, tears, urine, nasal mucus and skin secretions need to be carefully investigated. An additional prospect would be to find the similarities between eccDNA-mediated drug resistance in cancer cells and pesticide resistance in plant systems. This perspective will help to decode the use of small RNA regulatory sequences in plant sources as anticancer agents against cancer cells displaying drug resistance due to abundance of eccDNAs.

## Conclusion

The genomic architectures of living systems including plants, yeasts and animals are known to display genomic instability and display eccDNAs generated from chromosomal DNA. There is a need for basic and preclinical studies examining the basis of formation of eccDNAs within the mammalian, plant and yeast system, and further studies are required to establish their connection with stress, aging, environmental pressure, abnormalities and cancerous nature of normal cells. Furthermore, the high abundance and varied nature of eccDNAs with mutations in cancer cells and cancer-associated cells suggest a high potential to drive drug resistance, metastasis and angiogenesis. Increased research on the role of eccDNAs in tumor heterogeneity, drug resistance and biomarker discovery, bringing a new area ‘Circulomics’ in cancer biology, is warranted using the cutting edge tools available in the genomics and bioinformatics fields. Taken together, investigations at basic, preclinical and clinical levels including development of therapeutics and diagnostics in cancer have potential to result in precise and personalized drugs in the form of gene therapy, small RNA mimetics and pharmacological inhibitors.

**Executive summary**

- Tumor heterogeneity is driven by various factors including genetics, epigenetics and distinct cellular components including immune cells, stromal cells and microbiotas in the local niche.
- Besides the various molecular components contributing to intratumor heterogeneity, heterogeneous availability of extrachromosomal circular DNAs (eccDNAs) inside cells and in the extracellular milieu provides another dimension and adds to the complex nature of tumor microenvironment.
- With regards to translational biology, approaches to reduce the level of eccDNAs appear promising for the future.
- A potential use of eccDNAs as biomarkers for the early detection of cancer is foreseeable, specifically in noninvasive methods using body fluids including serum, urine, saliva, tears, nasal mucus and skin secretions.
- The eccDNAs are proposed to serve as the key paracrine signaling messengers from the primary tumor sites to secondary metastatic tumor sites.
